# Utility of Two PANSS 5-Factor Models for Assessing Psychosocial Outcomes in Clinical Programs for Persons with Schizophrenia

**DOI:** 10.1155/2013/705631

**Published:** 2013-12-05

**Authors:** Jeanette M. Jerrell, Stephanie Hrisko

**Affiliations:** Department of Neuropsychiatry and Behavioral Science, University of South Carolina School of Medicine, 3555 Harden Street Extension, 15 MP, Suite 301, Columbia, SC 29203, USA

## Abstract

Using symptom factors derived from two models of the Positive and Negative Syndrome Scale (PANSS) as covariates, change over time in consumer psychosocial functioning, medication adherence/compliance, and treatment satisfaction outcomes are compared based on a randomized, controlled trial assessing the effectiveness of antipsychotic medications for 108 individuals diagnosed with schizophrenia. Random effects regression analysis was used to determine the relative performance of these two 5-factor models as covariates in estimating change over time and the goodness of fit of the regression equations for each outcome. Self-reported psychosocial functioning was significantly associated with the relief of positive and negative syndromes, whereas patient satisfaction was more closely and significantly associated with control of excited/activation symptoms. Interviewer-rated psychosocial functioning was significantly associated with relief of positive and negative symptoms, as well as excited/activation and disoriented/autistic preoccupation symptoms. The VDG 5-factor model of the PANSS represents the best “goodness of fit” model for assessing symptom-related change associated with improved psychosocial outcomes and functional recovery. Five-factor models of the syndromes of schizophrenia, as assessed using the PANSS, are differentially valuable in determining the predictors of psychosocial and satisfaction changes over time, but not of improved medication adherence/compliance.

## 1. Introduction

Recovery from a serious mental illness is now widely endorsed as a guiding principle of mental health policy and brings new rules for services, for example, user involvement and person-centered care, as well as participatory approaches to evidence-based medicine and policy. Recovery is determined to varying degrees by the severity of psychopathology and cognitive deficits, objective social variables such as marital status and living situation, previous psychosocial functioning, course of illness variables such as age of onset or duration, and personal risk factors such as gender or race [1, 2]. Cognitive function, encompassing all aspects of perceiving, thinking, reasoning, and remembering, is a key predictor of functional recovery [3].

Whether pharmacotherapy or psychosocial interventions are being investigated, treatment studies for persons with schizophrenia typically encompass a range of outcomes, including symptoms, psychosocial functioning, quality of life, treatment adherence or compliance, or satisfaction with the treatment received [2, 4]. Previous research has demonstrated that these outcome dimensions are significantly interrelated [5, 6]. Available, effective pharmacological treatments, psychological therapies, and rehabilitation strategies may achieve significant symptom relief but may not improve social functioning or achieve functional recovery [3, 4, 6]. Perceived patient satisfaction with treatment is positively and closely associated with medication compliance, symptom improvement, and quality of life/psychosocial functioning [5, 6].

The Positive and Negative Syndrome Scale (PANSS) is the most widely-used outcome instrument in randomized, controlled trials for assessing the effects of antipsychotic medication, and other treatments on the symptoms of schizophrenia. Five-factor models for interpreting the PANSS are thought to be more representative of the syndromes of schizophrenia [7–9] than the original 3 subscales: positive, negative, and psychopathology [10], especially in a chronic course [11]. These five factors remain consistent whether patients are on or off medication [7, 12] and are consistently identified across subgroups of patients to include negative, positive, disorganized/autistic preoccupation, excited/activation (hostility/aggression), and dysphoric mood/emotional distress (depression) [7–9, 11–13].

Liu-Seifert et al. [14] suggested that a 5-factor model captures specific syndromes (i.e., the positive, hostility/excitement and depression factors) which influence whether or not patients discontinue their prescribed medication (adherence). Furthermore, Gharabawi et al. [15] found that medication satisfaction, which is highly associated with treatment compliance, is correlated with an improvement in the hostility/excited/activation, positive, and anxiety/depression/emotional distress factors on the PANSS. Since autistic preoccupation/disorganized symptoms may be more important than positive or negative symptoms in predicting functional outcomes because they negatively impact the ability to work, performing daily living activities, or social functioning, they are also a significant factor to consider in measuring treatment outcomes and medication efficacy [1]. However, research to date focused on the disorganized/autistic preoccupation dimension of schizophrenia and its relationship with psychosocial functioning has been very limited.

Moreover, controversy persists about which 5-factor model best captures the syndromes of schizophrenia for use in research and clinical practice, since the existing literature lacks direct performance comparisons among any of the 5-factor models beyond confirmatory factor analysis [7, 16]. To our knowledge, there have been no studies comparing the Pentagonal (PM) [7] and Van der Gaag (VDG) [16] 5-factor models on a broader range of statistical criteria for their utility in predicting change over time in psychosocial functioning, medication adherence/compliance, and treatment satisfaction, which is the objective of the analyses presented herein.

## 2. Materials and Methods

The data employed in this reanalysis were collected using a 12-month, prospective, open-label, randomized, controlled trial design in a public mental health system [17]. At study entry, adult patients with a primary diagnosis of schizophrenia or schizoaffective disorder using DSM IV criteria, two or more acute psychiatric hospitalizations within the prior 12 months, being noncompliant with outpatient treatment, and not taking a novel antipsychotic medication for six to eight weeks or more during the three months prior to an index hospitalization were recruited into this study at two acute care psychiatric hospitals. Exclusion criteria were having a DSM IV primary diagnosis of organic brain syndrome, mental retardation, or a primary substance-related disorder, as determined by the treating psychiatrist either in the index hospitalization or in outpatient maintenance treatment. Patients were asked to participate in the study in an assigned medication group to comply with Institutional Review Board requirements and the clinical standards of the two hospitals. All study patients provided written, informed consent to participate in the research as well as consent to be treated with the assigned/prescribed antipsychotic medication. Patients were then interviewed (i.e., asked to recall their symptoms and rate their psychosocial functioning during the three months prior to the interview) at baseline and at 3-month intervals for up to 12 months after study enrollment (five occasions) [17].

### 2.1. Measures

The standardized, published outcome instruments employed in this study were the Positive and Negative Syndrome Scale (PANSS) [13]; Role Functioning Scale (RFS) [18]; Social Adjustment Scale—Severely Mentally Ill (SAS-SMI) [19]; and Client Satisfaction Questionnaire-8 (CSQ-8) [20]. Both the RFS and the SAS-SMI are scored for relationships with family and friends, involvement in work or productive activities, and involvement in community services/activities. All interviewers received specialized training in performing the PANSS ratings, had training and experience in previous clinical trials using the other psychosocial functioning instruments, and had experience in maintaining interrater reliability [17]. Indicators of pharmacotherapy practice, adherence, and compliance, extracted from medical record reviews for each patient for each time period, entailed days per occasion the patient was taking the assigned medication (adherence) and ratings of the percentage of time per occasion the patient was compliant with their prescribed medication regimen (i.e., getting regular injections, taking oral medications as prescribed, getting refills on time, etc.) or was prescribed an anticholinergic medication for extrapyramidal side effects. “Number of lifetime hospitalizations” and “total number of days of acute hospitalization in the prior six months” were used as proxies for chronicity (long-term severity) or acuity (recent severity) in the randomization procedure and in the analyses [17].

### 2.2. Data Analyses

PANSS item scoring procedures for the PM and VDG 5-factor models were based on previously published reports [16, 21]. Data for the RFS, SAS-SMI, adherence, compliance, and CSQ-8 study analyses (*N* = 108) were analyzed using the SAS PROC MIXED facility for repeated measures designs. Preliminary analyses where initially conducted for each outcome to assess and select the best covariance structure for each analysis. All outcome estimates were computed via a restricted likelihood method. Seven time-invariant covariates related to sociodemographic risk factors (gender, race, and age 18–36 years at baseline measurement), severity (>7 prior inpatient admissions, >4 inpatient days in the six months prior to baseline), and assigned medication group, the principal covariate of interest, were initially included in all models. Three time-dependent covariates (time period, anticholinergic medication use, and medication compliance) were included in all models (except medication compliance with itself), with time period as the principal covariate of interest. In addition, four combinations of two or three time-dependent covariates were included (PM Negative and PM Positive scores; PM Activation, PM Dysphoric Mood and PM Autistic Preoccupation score; VDG Positive and VDG Negative scores; and VDG Disorganized, VDG Excited, and VDG Emotion scores), depending on the regression model under review. These variations were due to moderate correlations between the disorganized/autistic preoccupation and positive syndrome factors in each model which indicated some collinearity. Covariates with *P* values >0.05 were removed one at a time from the model in a stepwise manner until only those covariates with *P* values ≤0.05 remained in the final model. All post hoc testing employed the Tukey-Kramer adjustment to protect the overall 0.05 significance level, and all statistical analyses were performed using SAS software, version 9.2 (SAS Institute, Cary, North Carolina, USA).

To estimate and compare the “goodness of fit” for each mixed model regression calculated, the Akaike Information Criteria (AIC) statistic for each model was used [22, 23]. This statistic may be conceptualized as a measure of the distance between two density functions (i.e., each regression equation and an estimated “true” equation) [24]. Based on the AIC values obtained for each final, reduced equation (i.e., only the significant covariates were included), the model with the smallest value of AIC is considered to be the “best fit.”

## 3. Results

The final study sample of 108 patients was comprised as follows: 63% were male, 73% were non-White, and 67% were diagnosed with schizophrenia (versus schizoaffective disorder) (Table 1). The mean age of these patients was 37 years. On average, patients had 13 days of acute hospitalization in the six months prior to participating in this study and 10 prior admissions to acute psychiatric care. Most (52.8%) had less than a high school education, and the mean length of illness (since first hospitalization in this mental health system) was 11.6 years.

PANSS items on the PM and VDG factors are compared in Table 2. There is a difference in the specific items comprising each factor dyad of the two PANSS models compared in this study, that is, Positive, Negative, Disorganized/Autistic Preoccupation, Excitement/Activation, and Dysphoric Mood/Emotional Distress. The VDG factors include more items and there are more overlapping items across factors, for example, on both the Positive and Disorganized/Autistic Preoccupation factors, symptoms of “lack of insight and judgment,” and “difficulty in abstracting” are included, whereas “active social avoidance” is included on 4 of the 5 VDG factors. Although some investigators have interpreted the Excitement/Activation and Dysphoric Mood/Emotional Distress factors derived from the PANSS as indicators of mania and depression [25, 26], such direct correlations were not confirmed in previous analyses of these data [27]. Descriptive data (means estimated from the random effects regressions, standard errors, and number of cases) for the RFS, SAS-SMI, adherence/compliance measures, and CSQ are presented in Table 3 by medication group.

### 3.1. Role Functioning (Interviewer-Rated Psychosocial Functioning)

While there was no indication of a medication group difference over time, there was a significant difference between treatment groups, with patients in the olanzapine group being rated higher on role functioning (across dimensions of relationships with family and friends, involvement in work or productive activities, and involvement in community services/activities) than those in the risperidone group, despite having significantly more days of hospitalization in the 6 months prior to entering the study after controlling for the significant effects of *decreasing* positive, negative, disorganized/autistic preoccupation, and excited/activation symptoms, and limited psychiatric chronicity (seven or fewer lifetime psychiatric hospital admissions) (Table 4).

### 3.2. Social Adjustment Schedule (Self-Reported Psychosocial Functioning)

The SAS-SMI total scores indicated that the self-reported psychosocial functioning (across dimensions of relationships with family and friends, involvement in work or productive activities, and involvement in community services/activities) of the conventional group increased over time, while that of the olanzapine and risperidone groups decreased over time (a statistically significant time x group interaction) after controlling for *decreasing *PM positive and negative symptoms and for *decreasing* PM activation and autistic preoccupation symptoms (Table 4). Furthermore, females expressed lower self-reported psychosocial functioning than males despite *decreasing* positive, negative, disorganized/autistic preoccupation, and excited/activation symptomatology.

### 3.3. Patient Satisfaction

The CSQ-8 client satisfaction scores indicated that *decreasing* negative symptoms, limited psychiatric chronicity (seven or fewer lifetime psychiatric hospital admissions), *increasing* medication compliance, and *decreasing* excited/activation symptoms were significantly associated with changes over time in client satisfaction (Table 4).

### 3.4. Adherence and Compliance

Adherence (days to discontinuation of the assigned medication) demonstrated a significant change over time by medication group after taking into account the significant influence of limited psychiatric acuity (less than 5 days of inpatient service in the 6 months prior to baseline), and *not* being prescribed an anticholinergic medication (i.e., not experiencing medication side effects) (Table 4). Adherence in the olanzapine group rose from an average of approximately 32 days at baseline to an average of 85 days at 6 months and stayed at that level until the 12-month occasion. None of the PM or VDG symptom covariates (positive, negative, disorganized/autistic preoccupation, or excited/activation) was significantly associated with this measure of adherence.

Patient compliance with medication ratings also demonstrated a significant change over time by medication group interaction after taking into account *not* using an anticholinergic medication or not experiencing any medication side effects (Table 4). Medication compliance in the olanzapine group rose from an average of 50%–75% of the time taking the medication as prescribed to an average of 75%–100% at the time of the 9- and 12-month record reviews. Again, none of the PM or VDG positive, negative, disorganized/autistic preoccupation, or excited/activation symptom covariates was significantly associated with changes in this measure of compliance.

### 3.5. Goodness of Fit

As shown in Table 4, a comparison of the final regression equations using the AIC statistic indicates that the VDG model consistently demonstrated the best “goodness of fit” for interviewer-rated psychosocial functioning (RFS) when positive and negative syndromes and medication compliance were taken into consideration. Furthermore, the VDG model demonstrated the best “goodness of fit” performance over time on all of the SAS-SMI analyses and on all of the CSQ analyses taking into consideration changes in positive, negative, disorganized/autistic preoccupation, or excited/activation syndromes. Since no PANSS symptom covariates were significantly associated with adherence or compliance, the “goodness of fit” AIC statistics are the same for all of these analyses.

## 4. Discussion

Self-reported psychosocial functioning on the SAS-SMI and interviewer-rated psychosocial functioning on the RFS were significantly associated with the relief of not only positive and negative syndromes, that is, the overt symptoms of psychosis, active social avoidance or withdrawal, blunted affect, impaired volition, and so forth, but also of the excited/activation syndrome, for example, hostility, aggression, impulsivity, tension, uncooperativeness, and the disorganized/autistic preoccupation syndrome, for example, inattentiveness, disorientation, disorganization, difficulty abstracting, and preoccupation. These results are consonant with previous studies indicating that psychosocial functioning is significantly associated with relief of positive and negative symptoms, with the control of excited/aggressive/hostile symptoms, and with improvement in the disorganized/autistic preoccupation factor [3, 6, 15, 16]. Moreover, relief of disorganized/autistic preoccupation symptoms is confirmed as an important component, along with positive or negative symptoms, in predicting functional outcomes, such as the ability to work, performing daily living activities, and social functioning, and appears to be a significant factor to consider in measuring treatment outcomes and medication efficacy, along with other symptom changes [1, 3]. Pharmacotherapy or psychosocial interventions targeted at relieving a broad array of the symptoms/syndromes of schizophrenia are perceived as improving self-reported or professionally rated psychosocial functioning and contributing to functional recovery.

Furthermore, patient satisfaction were significantly associated with control of excited/activation symptoms as well as relief from positive and negative syndromes. However, the dysphoric mood/emotional distress factor dyad from both models was not significantly related to any of the psychosocial functioning or patient satisfaction outcomes, which does not comport with the findings of Liu-Seifert et al. [14] or Gharabawi et al. [15].

Many investigators recognize that the number of factors used when analyzing the PANSS can impact the identification of subtypes of schizophrenia and/or the psychopathological processes underlying them, which may influence prognosis, therapeutic approaches, response to treatment, and prediction of related variables [7–9, 11–13]. Without using a 5-factor model of the PANSS to interpret findings, these aspects of schizophrenia would need to be measured with multiple assessment tools, thus increasing the potential response burden on patients. In this study, these two 5-factor models were used to determine the predictors associated with changes in psychosocial functioning, treatment adherence/compliance, and treatment satisfaction highlighted medication group differences that were not detected in the original analyses using the 3 subscales on the PANSS [17]. The olanzapine group displayed higher psychosocial functioning as rated by trained interviewers despite having significantly more days of acute hospitalization on entry into the study. Furthermore, medication adherence and compliance was significantly higher in the olanzapine group over time.

Neither adherence nor compliance was significantly associated with any type of symptom change in this cohort of patients, unlike the findings of Liu-Seifert et al. [14] and Gharabawi et al. [15], but both adherence and compliance were significantly associated with a lack of extrapyramidal medication side effects or not taking anticholinergic medications for medication side effects. This lack of association with symptom relief may indicate that adherence/compliance is more directly connected to perceived medication side effects than to perceived symptom relief, or it could also be due to our adherence/compliance measures not being broad enough or precise enough to capture the same aspects as assessed by other investigators [28] although both subjective and objective measures of adherence and compliance were employed. Nonetheless, based on these results, clinical service programs should focus on minimizing the neurological, metabolic, or other adverse effects related to psychotropic medication use to ensure patient adherence and compliance.

Finally, across most outcome dimensions of psychosocial functioning and consumer satisfaction assessed herein, the VDG 5-factor model demonstrated the best “goodness of fit” performance over time on the RFS and all of the SAS-SMI and CSQ analyses taking into consideration changes in positive, negative, disorganized/autistic preoccupation, and excited/activation syndromes. Since the choice of a syndrome factor model may influence our understanding of outcomes and intervention effectiveness for people with schizophrenia, our recommendation concerning a model of choice is guided not only by statistical criteria but also by the broader set of symptoms captured within each VDG factor, thus allowing clinicians and consumers to more comprehensively assess their relief/remission. Furthermore, flexibility in the factor model chosen is also prudent because we have previously demonstrated several important performance differences between the PM and VDG models in their representation of changes-over-time using the syndromes of schizophrenia as the outcomes of interest, with all five PM model factors demonstrating the best “goodness of fit” [27].

These findings are particularly important given the current clinical emphasis on evidence-based, multifaceted therapeutic approaches [4], especially those which integrate cognitive remediation with social skills therapies to achieve not only symptom remission but also functional recovery in patients with schizophrenia [3]. Effectiveness studies of the Integrated Psychological Therapy program have demonstrated significant improvements in the original 3 subscales of the PANSS, as well as on psychosocial functioning, social cognition, and neurocognition instruments [3]. It would be very interesting to see if there is change in the VDG Disorganized factor over time in a reanalysis of their data. Such a reanalysis would provide additional findings focused on the disorganized/autistic preoccupation syndrome of schizophrenia and its relationship with psychosocial functioning.

## 5. Conclusion

Based on these results, the VDG 5-factor model of the PANSS is potentially the most useful for assessing symptom-related change on the positive, negative, disorganized/autistic preoccupation, and excited/activation factors associated with improved psychosocial outcomes and functional recovery in routine practice settings as well as in randomized, controlled trials. Furthermore, outcome studies in routine or controlled trial settings should consistently assess changes on the positive, negative, disorganized/autistic preoccupation, and excited/activation PANSS factors to more comprehensively determine their association with improved psychosocial outcomes and functional recovery and to more effectively target interventions to consumer subgroups and maximize the effectiveness of these interventions.

## Figures and Tables

**Table 1:** Descriptive comparison at baseline.

Independent variable	Total (*N* = 108)	Olanzapine (*N* = 30)	Risperidone (*N* = 36)	Conventional (*N* = 42)	*P* value*
Ethnicity: nonwhite	79 (73.1)	19 (63.3)	28 (77.8)	32 (76.2)	0.36
Gender: male	68 (63.0)	17 (56.7)	23 (63.9)	28 (66.7)	0.68
Diagnosis: schizoaffective disorder	36 (33.3)	12 (40.0)	11 (30.6)	13 (31.0)	0.66
Education level: <high school	70 (64.8)	20 (66.7)	23 (63.9)	27 (64.3)	0.97
Age	36.9 ± 8.8	34.1 ± 9.3	37.6 ± 9.2	38.4 ± 7.6	0.11
24 hr care days in past 6 months	12.6 ± 19.0	19.8 ± 25.3	11.1 ± 18.5	8.7 ± 11.9	0.04
Prior inpatient admissions	9.8 ± 8.2	9.4 ± 8.2	9.1 ± 7.6	10.5 ± 8.7	0.74
Taking anticholinergic meds	72 (66.7)	17 (56.7)	24 (66.7)	31 (73.8)	0.31

*Data shown are *N* (%) or mean ± SD.

**Table 2:** Item comparison for the PM and VDG 5-factor models.

Factor represented	Pentagonal model	Van der Gaag's model
Positive	Delusions Unusual thought content Grandiosity Hallucinations Somatic concern	Delusions Hallucinations Unusual thought content Suspiciousness Grandiosity Somatic concern Active social avoidance Lack of judgment and insight Difficulty in abstraction (−)

Negative	Lack of spontaneity Blunted affect Emotional withdrawal Poor rapport Apathetic social withdrawal Motor retardation Mannerisms Uncooperativeness Impaired volition Impulsivity	Lack of spontaneity Blunted affect Emotional withdrawal Poor rapport Apathetic social withdrawal Motor retardation Active social avoidance Uncooperativeness Impaired volition Conceptual disorganization (−)

Disorganized/autistic preoccupation	Poor attention Preoccupation Difficulty in abstraction Stereotyped thinking Disturbed volition Hallucinations	Stereotyped thinking Poor attention Disorientation Conceptual disorganization Difficulty in abstraction Mannerisms Lack of judgment and insight Disturbed volition Preoccupation Unusual thought content

Excited/activation	Hostility Impulsivity Excitement Uncooperativeness Poor rapport Tension	Hostility Impulsivity Excitement Uncooperativeness Grandiosity Poor rapport Tension Active social avoidance

Dysphoric mood/ emotional distress	Anxiety Tension Guilt Depression Somatic concern	Anxiety Tension Guilt Depression Somatic concern Suspiciousness Active social avoidance Preoccupation

**Table 3:** Estimated means and standard errors for RFS, SAS, and CSQ measures by medication group and time period.

	Baseline mean (SE) ± SD	3 months mean (SE) ± SD	6 months mean (SE) ± SD	9 months mean (SE) ± SD	12 months mean (SE) ± SD
OLANZAPINE (*N* = 30)					
RFS total SAS-SMI total CSQ-8 total Adherence Compliance	2.31 (0.21) 67.26 (3.72) 21.23 (1.19) 36.43 ± 36.27 3.57 ± 0.79	3.33 (0.24) 79.36 (4.50) 21.30 (1.48) 70.86 ± 26.84 3.56 ± 0.65	3.50 (0.30) 61.60 (5.79) 24.71 (2.14) 87.43 ± 14.27 3.53 ± 0.66	3.51 (0.27) 72.09 (4.95) 22.97 (1.79) 83.52 ± 22.52 3.62 ± 0.59	3.54 (0.26) 71.25 (4.69) 23.97 (1.66) 81.16 ± 24.27 3.68 ± 0.58

RISPERIDONE (*N* = 36)					
RFS total SAS-SMI total CSQ-8 total Adherence Compliance	2.17 (0.19) 59.45 (3.40) 21.20 (1.10) 44.93 ± 39.04 3.75 ± 0.58	2.66 (0.24) 64.53 (4.46) 25.65 (1.49) 71.69 ± 25.70 3.43 ± 0.75	2.67 (0.25) 60.65 (4.82) 24.48 (1.81) 71.68 ± 28.15 3.25 ± 0.88	2.61 (0.25) 63.10 (4.64) 21.70 (1.57) 80.48 ± 18.72 3.16 ± 0.79	2.52 (0.25) 59.40 (4.64) 19.90 (1.83) 69.42 ± 25.78 3.28 ± 0.71

CONVENTIONAL (*N* = 42)					
RFS total SAS-SMI total CSQ-8 total Adherence Compliance	2.48 (0.18) 67.64 (3.14) 22.24 (1.02) 75.28 ± 27.53 2.65 ± 0.83	2.75 (0.24) 60.63 (4.33) 25.93 (1.63) 72.24 ± 27.89 3.21 ± 0.85	2.98 (0.29) 65.66 (5.61) 25.55 (2.30) 77.68 ± 28.04 3.32 ± 0.79	3.21 (0.27) 78.73 (5.13) 27.08 (1.74) 72.90 ± 28.85 3.23 ± 0.84	3.11 (0.27) 77.43 (5.08) 25.93 (1.83) 77.28 ± 27.11 3.28 ± 0.84

**Table 4:** Random effects regression results.

Variable	*F*	*P*	AIC^a^
RFS total—with PM covariates			1542.1
Treatment group	6.10	0.003	
Occasion	2.58	0.02	
Less than 8 prior admissions	6.73	0.01	
PANSS PM negative score	23.34	<0.0001	
PANSS PM positive score	23.42	<0.0001	

RFS total—with VDG covariates			1538.8
Treatment group	6.40	0.002	
Occasion	2.32	0.03	
Less than 8 prior admissions	5.91	0.02	
PANSS VDG negative score	6.72	0.01	
PANSS VDG positive score	44.14	<0.0001	

RFS total—with PM covariates			1484.0
Treatment group	5.40	0.006	
Occasion	5.91	<0.0001	
Less than 8 prior admissions	6.32	0.01	
Medication compliance	5.31	0.02	
PANSS PM activation score	8.75	0.003	
PANSS PM autistic preoccupation	24.86	<0.0001	

RFS total—with VDG covariates			1520.1
Treatment group	6.26	0.002	
Occasion	4.01	0.0008	
Less than 8 prior admissions	6.04	0.02	
PANSS VDG disorganized score	36.78	<0.0001	
PANSS VDG excited score	5.41	0.02	

SAS-SMI total—with PM covariates			2431.1
Female	3.12	0.08	
PANSS PM negative score	17.45	<0.0001	
PANSS PM positive score	14.50	0.0002	

SAS-SMI total—with VDG covariates			2399.7
Female	6.02	0.02	
No schizoaffective diagnosis	4.84	0.03	
Less than 8 prior inpatient admissions	4.46	0.04	
PANSS VDG negative score	11.93	0.0006	
PANSS VDG positive score	26.63	<0.0001	

SAS-SMI total—with PM covariates			2430.1
Treatment group x occasion	1.89	0.04	
PANSS PM activation score	7.05	0.008	
PANSS PM autistic preoccupation score	7.07	0.008	

SAS-SMI total—with VDG covariates			2416.1
Treatment group	2.33	0.01	
PANSS VDG disorganized score	5.21	0.02	
PANSS VDG excited score	10.69	0.001	

CSQ total—with PM covariates			1531.8
Less than 8 prior inpatient admissions	8.92	0.004	
PANSS PM negative score	12.18	0.0006	

CSQ total—with VDG covariates			1523.4
Less than 8 prior inpatient admissions	7.13	0.009	
PANSS VDG negative score	4.31	0.04	
PANSS VDG positive score	14.07	0.0002	

CSQ total—with PM covariates			1475.1
Less than 8 Prior inpatient admissions	8.35	0.005	
Medication compliance	4.62	0.03	
PANSS PM activation score	25.88	<0.0001	

CSQ total—with VDG covariates			1474.6
Less than 8 prior inpatient admissions	8.72	0.004	
Medication compliance	4.27	0.04	
PANSS VDG excited score	27.76	<0.0001	

Adherence—with PM covariates			3425.8
Occasion	5.22	<0.0001	
Treatment group x occasion	3.34	0.0004	
Less than 5 inpt days in 6 months prior to baseline	5.66	0.02	
No anticholinergic use (Cogentin)	4.59	0.03	

Adherence—with VDG covariates			3425.8
Occasion	5.22	<0.0001	
Treatment group x occasion	3.34	0.0004	
Less than 5 inpt days in 6 months prior to baseline	5.66	0.02	
No anticholinergic use (cogentin)	4.59	0.03	

Compliance—with PM covariates			702.1
Occasion	8.36	<0.0001	
Treatment group x occasion	2.42	0.0007	
No anticholinergic use (cogentin)	7.53	0.006	

Compliance—with VDG covariates			702.1
Occasion	8.36	<0.0001	
Treatment group x occasion	2.42	0.0007	
No anticholinergic use (cogentin)	7.53	0.006	

^a^Akaike information criteria.
